# From Genes to Imaging Phenotypes: Radiomics and Machine Learning as Tools to Decode Molecular Pathways in Alzheimer’s Disease

**DOI:** 10.3390/genes17060672

**Published:** 2026-06-08

**Authors:** Marta Rusek, Monika Pitucha

**Affiliations:** Independent Unit of Radiopharmacy, Department of Organic Chemistry, Faculty of Pharmacy, Medical University of Lublin, 4a Chodźki Street, 20-093 Lublin, Poland; monika.pitucha@umlub.edu.pl

**Keywords:** Alzheimer’s disease, radiomics, machine learning, molecular pathways, genetics, PET imaging, tau, amyloid, precision medicine, neurodegeneration

## Abstract

Alzheimer’s disease (AD) is a heterogeneous neurodegenerative disorder driven by complex interactions between genetic susceptibility, molecular pathways, and progressive brain alterations. Key genetic factors, including *APOE*, *TREM2*, and *MAPT*, contribute to pathological processes such as amyloid-β accumulation, tau aggregation, neuroinflammation, and synaptic dysfunction. Despite substantial advances in understanding these mechanisms, translating molecular insights into clinically accessible biomarkers remains a major challenge. Radiomics and machine learning (ML) have emerged as promising approaches for extracting high-dimensional quantitative features from medical imaging data and identifying complex patterns associated with disease processes. Radiomic features capture spatial heterogeneity and subtle characteristics of neurodegeneration that are not discernible using conventional imaging analysis. When integrated with ML, these features may serve as noninvasive surrogates of molecular activity, enabling the identification of imaging signatures associated with specific genetic backgrounds and biological pathways. This review aims to explore how radiomics and ML can bridge the gap between genetic and molecular mechanisms and in vivo imaging phenotypes in AD. We summarize current knowledge on genetic determinants and molecular pathways and discuss advances in molecular imaging, particularly tracers targeting amyloid and tau pathology. Furthermore, we analyze the emerging role of radiomics and ML in linking imaging phenotypes with underlying biological processes. This integrative framework may support improved disease stratification, early diagnosis, and prediction of therapeutic response, contributing to the development of precision medicine strategies and future theranostic approaches in Alzheimer’s disease.

## 1. Introduction

Alzheimer’s disease (AD) is the most common cause of dementia worldwide and represents a major public health challenge [1]. It is a complex and multifactorial neurodegenerative disorder characterized by progressive cognitive decline, synaptic dysfunction, and neuronal loss [2]. Despite decades of research, the mechanisms underlying disease onset and progression remain incompletely understood, largely due to the interplay between genetics and environmental factors.

The pathogenesis of AD involves interactions between genetic susceptibility, molecular pathways, and environmental factors [3]. From a genetic perspective, mutations in *APP*, *PSEN1*, and *PSEN2* are linked to familial AD, whereas the *APOE ε4* allele is the strongest risk factor for sporadic AD [4,5,6]. In addition, genome-wide association studies (GWAS) have identified numerous susceptibility loci, including *TREM2*, which are implicated in immune regulation and microglial activation [7,8]. These genetic factors influence key biological processes, such as amyloid-β accumulation, tau pathology, and neuroinflammation [9,10]. At the molecular level, AD is characterized by a network of interconnected pathways rather than a single pathogenic cascade [11]. The classical amyloid hypothesis, centered on the accumulation of amyloid-β plaques, has been complemented by growing evidence highlighting the critical roles of tau pathology, mitochondrial dysfunction, oxidative stress, and dysregulated neuroinflammatory responses. Importantly, these processes do not occur in isolation but interact dynamically, contributing to the heterogeneity observed in clinical presentation and disease progression.

One of the major challenges in AD research is the translation of molecular and genetic insights into clinically actionable biomarkers. Traditional diagnostic approaches rely on cognitive assessment and structural imaging, which often detect changes only at advanced stages of the disease. In contrast, molecular imaging techniques, particularly positron emission tomography (PET) and magnetic resonance imaging (MRI), enable the in vivo visualization of these pathological processes at earlier stages [10,12,13,14]. However, conventional image interpretation remains largely qualitative or semi-quantitative, limiting its ability to fully capture disease complexity [15,16,17]. In this context, radiomics has emerged as a promising approach for extracting high-dimensional quantitative features from medical images, enabling the characterization of tissue heterogeneity and subtle spatial patterns [18,19]. When combined with machine learning, radiomics allows the identification of complex, nonlinear relationships within imaging data and facilitates the development of predictive and classification models [20]. Increasing evidence suggests that such imaging-derived features may effectively function as a form of “digital biopsy” [18,19,20,21] and may significantly improve diagnostic accuracy and prediction of disease progression compared to conventional imaging methods. By linking quantitative imaging features with specific biological mechanisms, this approach may enable a more precise characterization of disease subtypes, improve early diagnosis, and support the development of personalized therapeutic strategies [20]. Moreover, it aligns with the broader paradigm shift toward systems biology and precision medicine in neurodegenerative disorders.

Although numerous reviews have discussed molecular imaging, artificial intelligence, machine learning, or radiomics in Alzheimer’s disease separately, relatively few studies have attempted to integrate genetic susceptibility, molecular pathways, imaging-derived phenotypes, and computational analysis within a unified translational framework. Given the multifactorial and biologically heterogeneous nature of AD, such integration may provide a more comprehensive understanding of disease mechanisms and facilitate the development of biologically informed precision medicine strategies. In this context, radiomics and machine learning should be considered emerging and still evolving methodologies rather than fully established clinical tools. While these approaches have demonstrated promising potential for identifying complex imaging patterns and supporting multimodal disease characterization, several important methodological and translational challenges remain. These include variability in imaging acquisition and preprocessing protocols, radiomic feature instability, limited external validation, risk of overfitting in small datasets, and incomplete biological interpretation of imaging-derived features. Addressing these limitations will be essential for the future clinical translation of imaging-based computational biomarkers in Alzheimer’s disease.

Therefore, this review aims to provide a comprehensive overview of current knowledge on the genetic background and molecular pathways of AD and to explore the emerging role of radiomics and machine learning in translating these insights into clinically relevant imaging biomarkers. We further discuss the potential of this integrative framework that may contribute to improved disease stratification, earlier diagnosis, and prediction of therapeutic response while supporting future precision medicine and theranostic approaches in Alzheimer’s disease.

## 2. Genetic Background of Alzheimer’s Disease

Alzheimer’s disease (AD) has a substantial genetic component that includes both rare highly penetrant mutations and common susceptibility variants contributing to disease risk. Importantly, the genetic architecture of AD is heterogeneous and includes distinct categories of genetic determinants that differ in effect size, biological relevance, and clinical implications [6]. Early-onset familial Alzheimer’s disease (EOFAD), accounting for less than 5% of cases, is primarily associated with highly penetrant autosomal dominant mutations in the amyloid precursor protein (*APP*), presenilin 1 (*PSEN1*), and presenilin 2 (*PSEN2*) genes. These mutations directly affect amyloid precursor protein processing and promote increased production of the aggregation-prone amyloid-β42 isoform, leading to early and relatively predictable disease onset. Consequently, carriers of these mutations typically develop AD at a younger age, often before the age of 65 years, with a relatively predictable course [4,6].

In contrast, late-onset Alzheimer’s disease (LOAD), which represents the vast majority of AD cases, is polygenic and influenced by numerous common and rare susceptibility variants. The apolipoprotein E (*APOE*) *ε4* allele remains the strongest common genetic risk factor for sporadic AD and is associated with increased disease risk and earlier onset in a dose-dependent manner [11]. However, unlike autosomal dominant mutations, *APOE ε4* acts as a risk modifier rather than a deterministic causal mutation. In addition to *APOE*, GWAS have identified numerous loci associated with immune regulation, lipid metabolism, endocytosis, synaptic function, and neuroinflammatory pathways, highlighting the complex and multifactorial genetic architecture of AD [22,23]. 

Among the rare risk variants associated with AD, triggering receptor expressed on myeloid cells 2 (*TREM2*) has attracted particular attention due to its role in microglial activation and innate immune regulation. *TREM2* variants are associated with increased AD risk and are thought to impair microglial responses to amyloid deposition and neuronal injury [24]. Other susceptibility genes identified through GWAS include clusterin (*CLU*), phosphatidylinositol binding clathrin assembly protein (PICALM), and complement receptor 1 (*CR1*), which are involved in lipid transport, endocytosis, and immune signaling pathways [22,23].

Tau biology also represents a major component of AD pathophysiology. Although mutations in the microtubule-associated protein tau (*MAPT*) gene are more strongly associated with frontotemporal lobar degeneration and related primary tauopathies than with typical AD, tau dysregulation and abnormal tau aggregation remain central pathological processes in AD [24]. Therefore, *MAPT*-related mechanisms remain highly relevant in the broader context of tau-mediated neurodegeneration and imaging-based phenotyping [24]. These genes converge on major pathways including amyloid processing, tau pathology, and neuroinflammation, highlighting the network-based nature of AD [25,26]. 

Recent advances in multi-omics approaches, including transcriptomics, proteomics, and epigenomics, have further expanded our understanding of AD genetics by revealing dynamic changes in gene expression and regulatory mechanisms. These studies emphasize that genetic risk is not solely determined by static DNA sequence variations but is also modulated by environmental influences and epigenetic modifications [22,23,27]. Taken together, the genetic landscape of AD underscores the importance of integrating genetic information with downstream molecular and phenotypic data. In this context, linking genetic determinants to in vivo imaging phenotypes represents a promising strategy for capturing the functional consequences of genetic variation and for advancing precision medicine approaches in AD. The increasing availability of multimodal datasets integrating genomics, transcriptomics, imaging, and clinical information has created new opportunities to investigate how genetic variability contributes to imaging-derived phenotypes and disease heterogeneity in Alzheimer’s disease.

## 3. Molecular Pathways in Alzheimer’s Disease

AD is driven by multiple interconnected molecular pathways rather than a single pathogenic mechanism [11]. While the classical amyloid cascade hypothesis has long dominated the field, it is now widely recognized that multiple biological processes including tau pathology, neuroinflammation, mitochondrial dysfunction, oxidative stress, and synaptic failure contribute to disease onset and progression [22]. These pathways interact dynamically, forming a multifactorial and heterogeneous landscape of neurodegeneration. Importantly, the molecular pathways involved in AD are highly interconnected rather than independent. Amyloid accumulation can trigger tau pathology and neuroinflammation, while inflammatory processes can, in turn, exacerbate amyloid deposition and tau propagation [9]. Similarly, mitochondrial dysfunction and oxidative stress interact with all major pathological processes, amplifying neuronal damage. In addition to classical pathways, emerging evidence highlights the role of impaired autophagy, dysregulated proteostasis, and cerebrovascular dysfunction in AD pathogenesis. These processes further contribute to protein aggregation and neuronal damage, reinforcing the multifactorial nature of the disease [5,28].

This network-based perspective emphasizes the need for integrative approaches that capture the complexity of AD biology. In this context, imaging-derived phenotypes, particularly those obtained through radiomics, may reflect the combined effects of multiple molecular processes rather than a single pathway. Such multidimensional signatures provide a promising avenue for linking molecular mechanisms with in vivo observations.

### 3.1. Amyloid Pathway

The amyloid-β (Aβ) pathway remains a central component of AD pathophysiology. Aβ peptides are generated through sequential proteolytic cleavage of the amyloid precursor protein by β-secretase and γ-secretase. This process produces Aβ isoforms of varying lengths, with Aβ42 being particularly prone to aggregation. The accumulation of Aβ leads to the formation of extracellular plaques, which are considered one of the pathological hallmarks of AD [29]. Beyond plaque formation, soluble Aβ oligomers are now recognized as highly neurotoxic species that impair synaptic function, disrupt calcium homeostasis, and trigger downstream pathological cascades. Importantly, Aβ accumulation is thought to act as an upstream event that initiates or accelerates other pathological processes, including tau pathology and neuroinflammation. Furthermore, recent therapeutic approaches targeting amyloid clearance have shown variable clinical efficacy, suggesting that amyloid accumulation alone may not fully explain disease progression [30].

### 3.2. Tau Pathology

Tau protein, encoded by the *MAPT* gene, plays a crucial role in stabilizing microtubules and maintaining neuronal structure. In AD, tau undergoes abnormal hyperphosphorylation, leading to its detachment from microtubules and subsequent aggregation into intracellular neurofibrillary tangles. The spread of pathological tau follows a characteristic pattern across brain regions, correlating closely with disease progression and cognitive decline [29,31,32,33]. Unlike amyloid pathology, tau accumulation shows a stronger association with neuronal loss and clinical symptoms [29,34]. Recent evidence suggests that tau pathology may propagate in a prion-like manner, spreading between neurons via trans-synaptic mechanisms. This supports the growing consensus that tau pathology is more closely associated with clinical symptoms than amyloid deposition.

### 3.3. Neuroinflammation

Neuroinflammation is increasingly recognized as a central driver of AD progression. Microglia, the resident immune cells of the central nervous system, play a dual role in maintaining homeostasis and responding to pathological insults. In the early stages of AD, microglia contribute to the clearance of Aβ deposits. However, chronic activation leads to a sustained inflammatory response that exacerbates neuronal damage. Microglial activation contributes to chronic inflammation and neuronal damage [10]. Chronic neuroinflammation is increasingly recognized as a key driver rather than a secondary consequence of neurodegeneration. Genetic factors such as *TREM2* highlight the importance of microglial function in AD [7,8]. Dysfunctional microglial responses may impair debris clearance while promoting the release of pro-inflammatory cytokines, thereby contributing to a toxic environment [35]. Astrocytes also participate in this process, further amplifying inflammatory signaling and disrupting neuronal support mechanisms [36,37,38,39].

### 3.4. Mitochondrial Dysfunction

Mitochondrial dysfunction is another critical component of AD pathogenesis. Neurons are highly energy-dependent cells, and impairments in mitochondrial function can lead to reduced ATP production, increased generation of reactive oxygen species (ROS), and activation of apoptotic pathways. Oxidative stress damages cellular components, including lipids, proteins, and DNA, further compromising neuronal viability. Notably, mitochondrial dysfunction and oxidative stress are closely linked with both amyloid and tau pathology, forming a feedback loop that accelerates neurodegeneration [40].

### 3.5. Synaptic Dysfunction

Synaptic loss is one of the earliest and most clinically relevant features of AD, strongly correlating with cognitive decline [41]. Aβ oligomers and pathological tau contribute to synaptic dysfunction by disrupting neurotransmission, impairing plasticity, and altering dendritic spine morphology. Over time, these changes lead to widespread neuronal loss and brain atrophy, particularly in regions critical for memory and cognition, such as the hippocampus and cortex. The progressive breakdown of neuronal networks ultimately underlies the clinical manifestations of AD [41,42]. The complexity and interdependence of these molecular pathways highlight the need for non-invasive tools capable of capturing their in vivo manifestations. Molecular imaging techniques, particularly PET and MRI, offer a unique opportunity to visualize and quantify these processes in the living brain [43,44,45,46].

## 4. Molecular Imaging in Alzheimer’s Disease

Molecular imaging has become an essential tool in AD research and clinical practice, enabling the in vivo visualization of pathological processes that were previously accessible only through post-mortem analysis. In particular, PET and MRI techniques provide complementary insights into the structural, functional, and molecular alterations associated with neurodegeneration [47,48]. These modalities play a crucial role in bridging the gap between molecular mechanisms and observable disease phenotypes. Amyloid tracers such as PiB and florbetapir detect plaque deposition while tau PET provides insights into neurofibrillary pathology, whereas FDG-PET reflects metabolic dysfunction [43,49,50,51], and MRI reveals structural atrophy [45,46]. These modalities provide phenotypic representations of molecular processes but are limited by conventional analysis methods. Although amyloid PET enables early detection of pathological changes, its clinical utility is limited by the weak correlation between amyloid burden and cognitive impairment. In contrast, tau PET shows a stronger association with neurodegeneration and clinical symptoms, making it a more reliable biomarker for disease staging [34,52,53,54].

### 4.1. Amyloid PET Imaging

Amyloid PET imaging represents one of the most significant advances in AD diagnostics, allowing for the direct visualization of amyloid-β (Aβ) deposition in the brain. Radiotracers such as ^11^C-PiB (Pittsburgh Compound B) and ^18^F-labeled compounds including florbetapir, florbetaben, and flutemetamol selectively bind to fibrillar Aβ plaques, enabling their detection and quantification [55,56]. Amyloid PET has demonstrated high sensitivity for detecting early pathological changes, often preceding the onset of clinical symptoms by many years [57]. However, amyloid burden does not always correlate strongly with disease severity, as significant Aβ accumulation may also be observed in cognitively normal individuals [58]. This highlights the need for additional biomarkers that better reflect disease progression and neurodegeneration.

### 4.2. Tau PET Imaging

Tau PET imaging has emerged as a critical complement to amyloid imaging, providing insights into neurofibrillary tangle pathology. Radiotracers such as ^18^F-flortaucipir (AV-1451) enable the visualization of aggregated tau protein in specific brain regions [56,59]. Unlike amyloid deposition, tau accumulation shows a strong spatial and temporal correlation with neuronal loss and cognitive decline. The distribution of tau pathology follows well-characterized patterns such as Braak staging, making tau PET a valuable tool for disease staging and monitoring progression. Consequently, tau imaging is increasingly considered a more direct indicator of neurodegeneration than amyloid PET [60].

### 4.3. FDG-PET and Functional Imaging

Fluorodeoxyglucose (FDG)-PET provides information on cerebral glucose metabolism, serving as an indirect marker of neuronal activity and synaptic function. In AD, FDG-PET typically reveals hypometabolism in temporoparietal regions, the posterior cingulate cortex, and the precuneus [49,56]. Although FDG-PET does not target a specific molecular pathway, it reflects downstream functional consequences of pathological processes such as amyloid accumulation and tau-mediated neurodegeneration. As such, it offers valuable complementary information to amyloid and tau imaging.

### 4.4. Structural and Advanced MRI

Magnetic resonance imaging remains a cornerstone of AD evaluation, particularly for assessing structural changes. The most prominent MRI finding in AD is hippocampal atrophy, which correlates with memory impairment and disease progression. Cortical thinning and global brain atrophy are also commonly observed [45,61,62]. Beyond conventional structural imaging, advanced MRI techniques provide additional layers of information. Diffusion tensor imaging (DTI) enables the assessment of white matter integrity, while functional MRI (fMRI) captures alterations in brain connectivity and network organization. These modalities contribute to a more comprehensive understanding of neurodegenerative processes [45,61,62].

### 4.5. Imaging as a Surrogate of Molecular Pathways

Importantly, molecular imaging does not merely provide anatomical or functional information but reflects underlying biological processes. Amyloid PET captures Aβ deposition, tau PET visualizes neurofibrillary pathology, and FDG-PET reflects synaptic dysfunction [63,64]. Similarly, MRI-based measures of atrophy and connectivity represent the cumulative effects of multiple molecular pathways [65,66].

However, conventional analysis of imaging data is typically limited to global or regional summary metrics, such as standardized uptake value ratios (SUVRs) or volumetric measurements. These approaches may fail to capture the spatial heterogeneity and complexity of pathological processes in AD [67]. This limitation has driven the development of advanced computational approaches aimed at extracting more detailed and informative features from imaging data. In this context, radiomics has emerged as a powerful tool for quantifying imaging phenotypes at a much higher level of granularity.

## 5. Radiomics in Neurodegeneration

Radiomics is an emerging computational approach that enables the extraction of large numbers of quantitative imaging features from standard medical images, transforming imaging data into high-dimensional and analyzable datasets [15,16,17,20,21,68]. By converting imaging signals into quantitative descriptors, radiomics may provide additional information regarding tissue heterogeneity and spatial organization that is not readily appreciable through conventional visual assessment alone [17,20,69]. In neurodegenerative diseases, including Alzheimer’s disease, radiomics has attracted growing interest as a potential tool for identifying subtle imaging patterns associated with disease-related structural and molecular alterations.

However, despite its promising potential, the clinical translation of radiomics in Alzheimer’s disease remains limited. Variability in imaging acquisition protocols, reconstruction parameters, segmentation strategies, preprocessing pipelines, and feature extraction methods can substantially affect feature reproducibility and model generalizability. In addition, many published radiomics studies rely on relatively small and single-center datasets, increasing the risk of overfitting and limiting external validation. Consequently, although radiomics represents a promising research direction, many proposed imaging biomarkers remain investigational and require rigorous biological and multicenter validation before routine clinical implementation.

### 5.1. Radiomics Workflow

The radiomics pipeline typically consists of several sequential steps, including image acquisition, preprocessing, segmentation, feature extraction, and data analysis. Importantly, the reproducibility of radiomics studies depends heavily on methodological standardization across all stages of the workflow. Even minor variations in image acquisition, reconstruction algorithms, voxel size, segmentation approaches, or preprocessing techniques may significantly influence extracted radiomic features. For this reason, harmonization initiatives such as the Image Biomarker Standardization Initiative (IBSI) and multicenter validation studies are increasingly recognized as essential for improving reproducibility and facilitating future clinical translation [16,20,69].

### 5.2. Types of Radiomic Features

Radiomic features include intensity, shape, and texture descriptors that capture tissue heterogeneity [21,70]. First-order features describe the distribution of voxel intensities, such as mean, variance, skewness, reflecting basic signal characteristics. Also, shape-based features capture geometric properties of anatomical structures, such as volume, surface area, and compactness. In contrast, texture features quantify spatial relationships between voxels and provide information about tissue heterogeneity. These include metrics derived from gray-level co-occurrence matrices (GLCM), gray-level run-length matrices (GLRLM), and other statistical frameworks. Finally, higher-order features, obtained through the application of filters or transformations, enable the detection of more complex patterns [21,70]. These features collectively provide a multidimensional representation of imaging data, potentially reflecting underlying biological processes. Therefore, radiomics has been applied to MRI and PET to improve diagnosis and prediction [70]. However, reproducibility and standardization remain challenges [70].

Although these features may capture biologically relevant imaging heterogeneity, their direct biological interpretation remains challenging. Most radiomic descriptors are mathematically derived parameters, and only a limited number have been systematically correlated with histopathology, molecular biomarkers, or longitudinal clinical outcomes in AD. Therefore, caution is required when attributing specific biological meaning to individual radiomic features.

### 5.3. Applications for Alzheimer’s Disease

In the context of AD, radiomics has been applied to both MRI and PET imaging to improve disease characterization. Studies have demonstrated that radiomic features derived from structural MRI can capture patterns of cortical atrophy and hippocampal degeneration with greater sensitivity than traditional volumetric measures. Similarly, PET-based radiomics has been used to analyze the spatial distribution and heterogeneity of tracer uptake, particularly in amyloid and tau imaging. These approaches allow for a more nuanced assessment of pathological burden beyond global summary metrics.

**Figure 1:**
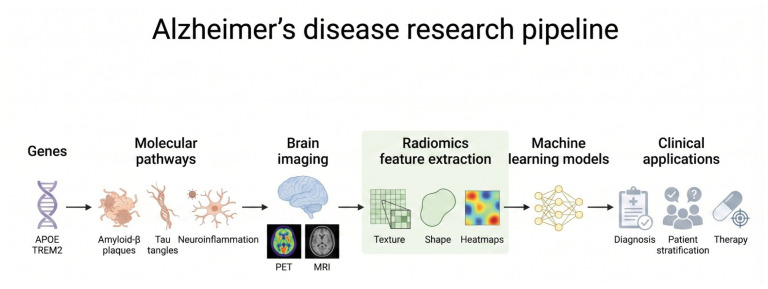
Conceptual schematic representation of a proposed integrative framework linking genetic determinants, molecular pathways, molecular imaging, radiomic feature extraction, and machine learning approaches in Alzheimer’s disease. The figure illustrates a hypothetical translational workflow intended to demonstrate potential relationships between biological mechanisms and imaging-derived phenotypes rather than an established clinical implementation pathway. Created in BioRender. Rusek, M. (2026) https://BioRender.com/x5y4rgv (accessed on 28 April 2026).

Radiomics has also shown promise in differentiating AD from other neurodegenerative disorders, identifying early-stage disease, and predicting cognitive decline. Importantly, these applications highlight the ability of radiomics to detect subtle imaging signatures that may precede overt clinical manifestations. As illustrated in Figure 1, the radiomics workflow in neurodegeneration involves several key steps, including image acquisition, preprocessing, segmentation, feature extraction, and data analysis using machine learning techniques. This standardized pipeline is essential for ensuring reproducibility and comparability across radiomics studies.

### 5.4. Representative Radiomics Studies in Alzheimer’s Disease

Several recent studies have demonstrated the potential utility of radiomics approaches in Alzheimer’s disease using MRI, PET, and multimodal imaging datasets. Structural MRI-based radiomics studies have frequently focused on the hippocampus, entorhinal cortex, and cortical gray matter regions, where texture and shape features have shown potential for distinguishing patients with AD from cognitively normal individuals and subjects with mild cognitive impairment (MCI). In some studies, radiomic texture features demonstrated improved sensitivity compared with conventional volumetric measures, particularly in detecting subtle structural heterogeneity associated with early neurodegeneration.

PET-based radiomics approaches have primarily investigated amyloid and FDG-PET imaging. Radiomic analysis of amyloid PET has been used to characterize spatial heterogeneity of tracer uptake beyond conventional standardized uptake value ratio (SUVR) measurements, while FDG-PET radiomics has been explored as a tool for identifying metabolic patterns associated with cognitive decline and disease progression. Several multimodal studies integrating MRI and PET radiomic features with clinical or cognitive data have reported improved classification performance compared with single-modality approaches.

More recently, imaging-genetics and multi-omics integration studies have begun exploring associations between radiomic signatures and genetic risk factors such as *APOE ε4* status. These approaches aim to identify imaging phenotypes potentially linked to underlying molecular mechanisms and biological heterogeneity. However, most currently available studies remain exploratory and are limited by relatively small sample sizes, methodological heterogeneity, lack of external validation, and inconsistent feature reproducibility. Consequently, although these findings are promising, further large-scale and longitudinal validation studies are necessary before radiomics-derived biomarkers can be considered clinically robust tools in Alzheimer’s disease. Representative applications of radiomics and machine learning in Alzheimer’s disease are summarized in Table 1. These studies illustrate the growing use of MRI, PET, and multimodal imaging approaches for disease classification, characterization of imaging heterogeneity, and integration with genetic and clinical data.

Despite encouraging findings, most currently available studies remain explorative and are characterized by methodological heterogeneity, limited external validation, and incomplete biological interpretation of imaging-derived features.

### 5.5. Radiomics as a Potential Non-Invasive Surrogate of Tissue Heterogeneity

One of the most frequently discussed concepts in radiomics is its potential role as a non-invasive surrogate of tissue heterogeneity or a form of “digital biopsy” [24]. In Alzheimer’s disease, this concept is particularly attractive because pathological processes such as amyloid deposition, tau aggregation, neuroinflammation, and synaptic dysfunction exhibit substantial spatial heterogeneity across brain regions. Quantitative imaging features may therefore provide indirect information regarding disease-related biological variability.

Nevertheless, the biological specificity of most radiomic features remains incompletely understood. Although some studies have reported correlations between imaging heterogeneity and molecular or pathological markers, direct biological validation remains limited. At present, radiomic features should therefore be interpreted primarily as imaging-derived mathematical descriptors that may correlate with underlying pathological processes rather than as direct measures of specific molecular mechanisms.

### 5.6. Reproducibility and Standardization Challenges

Despite its potential, radiomics faces several methodological challenges that must be addressed for clinical translation. Variability in image acquisition protocols, reconstruction parameters, and segmentation methods can significantly affect feature stability. Additionally, differences in feature extraction software and lack of standardized pipelines may limit reproducibility across studies [71]. Efforts such as the Image Biomarker Standardization Initiative (IBSI) aim to establish guidelines for radiomic feature definition and extraction. Furthermore, robust validation strategies, including external validation cohorts and cross-center studies, are essential to ensure the reliability of radiomics-based models [20,68,69,70,74].

While radiomics provides a powerful framework for quantitative feature extraction, the high dimensionality and complexity of the resulting data require advanced analytical methods for meaningful interpretation. Machine learning techniques offer a natural extension, enabling the identification of complex patterns and relationships within radiomic datasets [69].

Additional methodological concerns include feature instability across scanners and software platforms, segmentation variability, potential data leakage during model training, and insufficient use of external validation cohorts [71,74,75]. Furthermore, many machine learning models used in radiomics studies remain difficult to interpret, limiting biological explainability and reducing clinical trust [73,75]. Emerging approaches such as explainable artificial intelligence (XAI), harmonization methods (e.g., ComBat), and standardized reporting frameworks may help improve transparency and reproducibility in future studies [74,75].

## 6. Machine Learning and Data Integration

Machine learning (ML) has emerged as an important analytical framework for the analysis of high-dimensional biomedical datasets, particularly those generated through radiomics and multimodal imaging approaches [69]. By enabling the identification of complex and potentially non-linear relationships within large datasets, ML may facilitate the extraction of clinically and biologically relevant information from imaging-derived features. In Alzheimer’s disease, ML approaches have been investigated for disease classification, prediction of disease progression, patient stratification, and integration of multimodal data sets. Supervised methods (SVM, RF) and deep learning approaches are widely used in AD research [72,73]. The integration of imaging, genetics, and clinical data enhances predictive performance [73]. Key challenges include overfitting, limited dataset size, and model interpretability [71,75].

However, despite encouraging research findings, the clinical implementation of ML-based approaches in Alzheimer’s disease remains limited. Many published models are developed using relatively small, single-center, or highly curated datasets that may not adequately represent real-world clinical heterogeneity. Consequently, ML algorithms may inadvertently learn scanner-specific, protocol-specific, or cohort-specific characteristics rather than biologically meaningful disease patterns. These limitations raise important concerns regarding reproducibility, generalizability, and translational robustness.

### 6.1. Machine Learning Approaches in Alzheimer’s Disease

Machine learning techniques can be broadly divided into supervised, unsupervised, and deep learning methods. Supervised learning algorithms, such as support vector machines (SVM), random forests (RF), and logistic regression, are commonly used for classification and prediction tasks in AD, including distinguishing patients from healthy controls or identifying different disease stages [76]. Unsupervised learning approaches, including clustering and dimensionality reduction techniques, are applied to uncover hidden patterns and identify disease subtypes without predefined labels. These methods are particularly relevant for capturing the heterogeneity of AD [77,78].

Although deep learning approaches, particularly convolutional neural networks (CNNs), have demonstrated strong predictive performance in several neuroimaging studies, these models often function as “black boxes,” limiting interpretability and biological explainability [79]. This lack of transparency may complicate clinical adoption, especially in settings where understanding the rationale underlying model predictions is essential for clinical decision-making. As a result, explainable artificial intelligence (XAI) approaches are increasingly being explored to improve model transparency and interpretability.

### 6.2. Feature Selection and Dimensionality Reduction

One of the main challenges associated with radiomics is the high dimensionality of extracted features relative to the typically limited sample sizes. This imbalance increases the risk of overfitting and reduces model generalizability [80,81]. To address this issue, feature selection methods are employed to identify the most informative and stable features. Techniques such as recursive feature elimination, LASSO (least absolute shrinkage and selection operator), and principal component analysis (PCA) are commonly used to reduce dimensionality while preserving relevant information [71,74,82].

Inadequate feature selection strategies may increase the risk of model overfitting and lead to inflated performance estimates, particularly in studies with limited sample sizes. Furthermore, failure to separate training and validation procedures appropriately may result in data leakage, which can substantially compromise model reliability and reproducibility. Therefore, rigorous validation strategies, including independent external cohorts and cross-center validation, are essential for developing clinically meaningful predictive models.

### 6.3. Integration of Multimodal Data

A key strength of machine learning lies in its ability to integrate diverse data types into a unified analytical framework. In Alzheimer’s disease, this includes the combination of imaging data (PET, MRI), genetic information (e.g., *APOE*, *TREM2*), molecular and biochemical markers, and clinical and cognitive data [22,23,60,72]. Such multimodal integration enables a more comprehensive characterization of the disease, capturing interactions between genetic susceptibility, molecular pathways, and phenotypic manifestations [23,69,83]. Importantly, integrating imaging and genetic data may facilitate the identification of associations between specific genetic variants and distinct imaging phenotypes, thereby linking genotype to observable disease features.

Despite the conceptual advantages of multimodal integration, combining heterogeneous datasets presents substantial technical and methodological challenges. Differences in data quality, acquisition protocols, missing data structures, and cohort heterogeneity may complicate model development and reduce reproducibility. In addition, the increasing dimensionality associated with multimodal datasets may further increase the risk of overfitting if not appropriately controlled.

### 6.4. Predictive Modeling and Clinical Applications

Machine learning models trained on radiomic, and multimodal data have demonstrated potential in several clinically relevant applications. These include early detection of Alzheimer’s disease, differentiation between AD and other forms of dementia, prediction of disease progression, and assessment of therapeutic response [23,67,83].

In particular, ML-based models can identify subtle patterns that precede clinical symptoms, offering opportunities for earlier intervention. Additionally, predictive modeling may support patient stratification in clinical trials, improving the selection of individuals who are most likely to benefit from targeted therapies [67,72,73]. Despite promising results, several challenges limit the widespread clinical implementation of ML in AD research. These include small and heterogeneous datasets, lack of standardized methodologies, and limited external validation [72,73,75].

While machine learning enables the analysis and integration of complex datasets, its true value in Alzheimer’s disease lies in its ability to uncover relationships between imaging-derived features and underlying biological mechanisms. Although ML-based predictive models have shown promising results in research settings, relatively few have undergone rigorous prospective or multicenter validation. Consequently, most currently available models should still be considered investigational rather than clinically established tools. Future progress will require standardized methodological frameworks, larger longitudinal datasets, transparent reporting practices, and improved biological interpretability of model outputs.

Importantly, the value of machine learning in Alzheimer’s disease may ultimately depend less on maximizing predictive accuracy alone and more on improving biological understanding and clinically interpretable disease stratification. In this context, integrating imaging-derived features with molecular, genetic, and clinical information may provide a more biologically grounded framework for precision medicine approaches.

## 7. Linking Imaging Phenotypes with Molecular Pathways

A major challenge in Alzheimer’s disease research is establishing biologically meaningful links between genetic determinants, molecular pathways, imaging abnormalities, and clinical phenotypes. Although advances in genomics, molecular biology, and neuroimaging have substantially improved understanding of AD pathogenesis, translating these insights into clinically robust in vivo biomarkers remains difficult. In this context, radiomics and machine learning have emerged as promising investigational approaches for exploring relationships between imaging-derived phenotypes and underlying biological heterogeneity [15,17,68,81]. Genetic variants such as *APOE* influence imaging phenotypes, including amyloid burden and brain atrophy [84,85]. ML models integrating multimodal data enable genotype–phenotype mapping [68,72,73].

Nevertheless, it is important to emphasize that most currently identified associations between radiomic features and molecular mechanisms remain indirect and largely correlational. While imaging-derived features may capture biologically relevant spatial heterogeneity, definitive causal relationships between specific radiomic descriptors and particular molecular pathways have not yet been fully established. Consequently, imaging-genetic and imaging-molecular integration studies should currently be interpreted primarily as hypothesis-generating rather than definitive mechanistic frameworks.

Recent studies integrating imaging and genomics have demonstrated that specific genetic variants are associated with distinct imaging signatures, supporting the concept of imaging-genetics as a powerful tool for understanding disease heterogeneity [22,23,27,86]. However, the causal relationships between imaging-derived features and underlying molecular pathways remain incompletely understood.

### 7.1. Imaging Phenotypes as Surrogates of Molecular Processes

Molecular imaging modalities such as PET and MRI provide indirect but biologically informative representations of pathological processes occurring in Alzheimer’s disease. Amyloid PET reflects fibrillar amyloid-β deposition, tau PET captures regional tau accumulation, and MRI-derived structural measures reflect downstream neurodegenerative changes [45,49,63].

Radiomics extends this framework by quantifying spatial heterogeneity and imaging complexity beyond conventional regional summary metrics. For example, heterogeneous tracer uptake patterns observed in PET imaging may potentially correspond to differences in amyloid burden, tau propagation, metabolic dysfunction, or neuroinflammatory activity. Similarly, MRI-derived texture features may capture subtle microstructural alterations associated with neuronal degeneration or tissue disorganization. However, these associations remain incompletely understood, and most radiomic features lack direct histopathological validation. Therefore, radiomic descriptors should currently be interpreted as indirect imaging correlates of biological heterogeneity rather than direct measurements of specific molecular processes [17,68,80,81].

### 7.2. Linking Genetic Variability to Imaging Features

Emerging evidence suggests that genetic factors influence not only the risk of Alzheimer’s disease but also its phenotypic expression in the brain. Variants such as *APOE* ε4 have been associated with increased amyloid burden and distinct patterns of brain atrophy, while *TREM2* mutations are linked to altered microglial activity and neuroinflammatory responses [2,3,6].

Machine learning models integrating radiomic features with genetic data offer a novel approach to identifying associations between specific genetic variants and imaging phenotypes [68,69]. Such approaches may reveal imaging signatures that correspond to particular molecular pathways, effectively enabling the mapping of genotype to phenotype at the systems level.

Although initial imaging-genetics studies have reported associations between *APOE ε4* status and distinct imaging phenotypes, including increased amyloid burden and altered atrophy patterns, the reproducibility and biological specificity of these findings remain variable across studies. Furthermore, many currently available datasets remain insufficiently powered to fully capture the complexity of genotype–phenotype interactions in Alzheimer’s disease.

### 7.3. Capturing Pathway-Level Interactions Through Imaging

A key advantage of imaging-based approaches is their ability to reflect the combined effects of multiple molecular pathways. Unlike traditional biomarkers that often target a single molecule or process, imaging phenotypes capture the integrated outcome of interacting biological mechanisms [60,87]. For instance, regions exhibiting both amyloid accumulation and metabolic decline may reflect the interplay between amyloid toxicity and synaptic dysfunction. Similarly, patterns of tau spread observed in PET imaging may be influenced by both intrinsic neuronal vulnerability and extrinsic factors such as neuroinflammation [10,34,53,63].

Radiomics, by quantifying these spatial and textural patterns, provides a multidimensional representation of such interactions. When analyzed using machine learning, these features may help identify distinct disease subtypes or progression trajectories linked to specific pathway configurations [15,17].

Importantly, imaging-derived phenotypes likely reflect the combined downstream effects of multiple interacting biological processes rather than isolated molecular pathways. Consequently, interpreting radiomic signatures as representations of single pathological mechanisms may oversimplify the complex systems-level biology of Alzheimer’s disease. Instead, radiomics may provide a multidimensional representation of disease heterogeneity arising from the interaction of amyloid pathology, tau dysfunction, neuroinflammation, synaptic failure, vascular changes, and neurodegeneration.

### 7.4. Toward Imaging-Based Molecular Phenotyping

The integration of radiomics, machine learning, molecular imaging, and multi-omics data supports the emerging concept of imaging-based molecular phenotyping, in which patients may potentially be characterized according to underlying biological processes rather than solely clinical symptoms [68,86]. Although this framework remains largely investigational, it aligns with broader precision medicine efforts aimed at improving biologically informed disease stratification and individualized therapeutic approaches [86].

In Alzheimer’s disease, such phenotyping could facilitate the identification of individuals with predominant amyloid-driven pathology, tau-dominant neurodegeneration, or inflammation-associated disease progression. This distinction is particularly relevant in the context of emerging disease-modifying therapies targeting specific molecular mechanisms. However, substantial methodological, biological, and regulatory challenges must still be overcome before imaging-based molecular phenotyping can be translated into routine clinical practice.

### 7.5. Current Limitations and Research Gaps

Additional unresolved issues include limited biological interpretability of radiomic features, insufficient harmonization across imaging centers, and the lack of large-scale longitudinal multimodal datasets integrating imaging, genomics, transcriptomics, and neuropathological validation. Moreover, variability in segmentation methods and preprocessing pipelines may substantially influence reported imaging-genetic associations, complicating reproducibility across independent cohorts [22,27].

## 8. Clinical and Translational Implications

The integration of radiomics, molecular imaging, machine learning, and multi-omics data has the potential to improve the biological characterization of Alzheimer’s disease and support more individualized approaches to diagnosis and patient stratification. By capturing imaging-derived patterns that may reflect disease heterogeneity, these approaches could contribute to earlier detection of pathological changes and improved differentiation between biologically distinct disease subtypes.

However, despite encouraging research findings, most radiomics- and AI-based biomarkers in Alzheimer’s disease remain investigational and are not yet ready for routine clinical implementation. Significant challenges related to reproducibility, standardization, multicenter validation, biological interpretability, and regulatory approval must first be addressed before these approaches can be reliably integrated into clinical workflows.

Radiomic features extracted from PET and MRI data may reveal subtle alterations in brain structure and function before the onset of clinical symptoms [69,71]. When combined with ML, these features can be used to develop predictive models capable of identifying individuals at high risk of developing AD [72,73,76].

Given the substantial biological heterogeneity of AD, imaging-based molecular phenotyping may eventually support more precise patient stratification and improve the selection of individuals who are most likely to benefit from targeted therapeutic interventions. Nevertheless, further validation is required to determine whether currently identified radiomic signatures are sufficiently robust, reproducible, and biologically specific to support clinically actionable decision-making [23,83].

Furthermore, radiomics-based biomarkers may support monitoring of disease progression and therapeutic response [67,69]. Quantitative imaging features may serve as sensitive biomarkers for tracking changes over time, providing a more nuanced assessment than conventional clinical or imaging measures. This is particularly relevant in the context of emerging disease-modifying therapies targeting specific molecular mechanisms [23,83]. Importantly, longitudinal validation studies remain limited, and it is still unclear which radiomic features demonstrate sufficient temporal stability and sensitivity to serve as reliable biomarkers of disease progression or therapeutic response.

Although theranostic concepts remain at an early stage in neurodegenerative diseases compared with oncology, the integration of imaging-derived biomarkers with molecularly targeted therapies represents an important future research direction in Alzheimer’s disease [88,89,90].

## 9. Limitations and Challenges

Despite growing interest in radiomics and machine learning approaches in Alzheimer’s disease, substantial methodological, biological, and translational limitations currently restrict their broader clinical applicability. One of the most significant challenges is the lack of standardization across imaging acquisition protocols, reconstruction settings, segmentation methods, preprocessing pipelines, and feature extraction frameworks. These factors may substantially influence radiomic feature stability and reduce reproducibility across studies and imaging centers [71,72,81].

Another major limitation involves the relatively small size and limited diversity of many currently available datasets [22,25]. Single-center studies with highly selected cohorts may inadvertently introduce cohort-specific biases and increase the risk of overfitting. Furthermore, insufficient separation of training and validation procedures may lead to data leakage and artificially inflated model performance. Consequently, external validation using independent multicenter cohorts remains essential for assessing the robustness and generalizability of proposed imaging biomarkers.

The interpretability of machine learning models also remains a major challenge. Deep learning algorithms, while capable of high predictive performance, frequently operate as “black box” systems with limited biological transparency. This lack of explainability may reduce clinical trust and complicate regulatory approval. Emerging explainable artificial intelligence (XAI) approaches may help address some of these concerns by improving transparency and interpretability of model outputs [75,80].

Importantly, the biological meaning of many radiomic features remains incompletely understood. Although radiomic descriptors may capture imaging heterogeneity associated with disease-related processes, direct correlations with neuropathology, molecular biomarkers, or longitudinal clinical outcomes remain limited [22,75]. Additional multimodal validation studies integrating imaging, histopathology, fluid biomarkers, and genomics will therefore be necessary to establish biological relevance.

## 10. Future Perspectives

Future progress in Alzheimer’s disease research will likely depend on increasingly integrative and biologically informed analytical frameworks combining molecular imaging, radiomics, machine learning, genomics, transcriptomics, proteomics, and longitudinal clinical data [22,23]. Such multimodal approaches may improve understanding of disease heterogeneity and facilitate the development of more personalized diagnostic and therapeutic strategies [60].

Advances in artificial intelligence, particularly deep learning and explainable AI approaches, may improve the analysis of complex multimodal datasets and enhance model interpretability [62,72]. However, future development should prioritize not only predictive performance but also reproducibility, transparency, biological validation, and clinical utility [73,79].

In the long term, the integration of imaging-derived phenotypes with molecular and genetic information may contribute to the development of precision medicine strategies in Alzheimer’s disease. Nevertheless, substantial methodological, biological, and regulatory challenges remain before these approaches can be translated into routine clinical practice. Continued multicenter collaboration, harmonization of radiomics workflows, longitudinal validation studies, and integration with neuropathological and molecular data will be essential for future clinical implementation [22,59,60].

## Figures and Tables

**Table 1:** Representative radiomics and machine learning studies in Alzheimer’s disease using MRI, PET, and multimodal imaging approaches.

Study	Imaging Modality	Cohort/Objective	Brain Regions	Representative Features	ML Approach	Main Findings	Major Limitations
[69]	MRI radiomics	AD vs. controls	Cerebellar subregions	Texture and intensity features	ML multimodal classification	Improved diagnostic classification performance using cerebellar radiomics	Limited external validation and cohort size
[71]	MRI radiomics	Cognitive impairment in neurological diseases	Hippocampus and cortical regions	Texture heterogeneity and shape features	Multiple ML approaches	Radiomics as potential utility in cognitive impairment evaluation	Methodological heterogeneity across included studies
[67]	PET/SPECT imaging + AI	AD prediction	Whole-brain PET/SPECT regions	Uptake heterogeneity and texture features	AI-based predictive models	AI-enhanced PET imaging improved prediction of AD-related patterns	Limited standardization and reproducibility
[43]	PET molecular imaging	Molecular visualization in AD	Amyloid- and tau-positive cortical regions	Spatial uptake heterogeneity	Quantitative imaging analysis	PET imaging captures pathophysiological heterogeneity in AD	Limited biological specificity of imaging features
[62]	Multimodal MRI + wearable data	Early neurodegenerative disease detection	Whole-brain analyses	Combined multimodal imaging features	Multimodal machine learning	Improved diagnostic performance through multimodal integration	Increased dimensionality and overfitting risk
[72]	Multimodal AI	Systematic review of AD AI models	Whole-brain multimodal datasets	MRI, PET, and multimodal feature integration	Deep learning and multimodal AI	Multimodal AI approaches generally outperform single-modality models	Lack of harmonization and external validation
[46]	MRI deep learning	AD diagnosis and progression prediction	Cortical and hippocampal regions	Automatically learned hierarchical imaging features	Deep learning CNN framework	High classification accuracy for AD detection	Limited interpretability (“black box” issue)
[73]	Multimodal ML in neurodegeneration	Neurodegenerative disease diagnosis	MRI/PET multimodal analyses	Integrated imaging biomarkers	ML and deep learning	Multimodal markers improved classification performance	Dataset heterogeneity and reproducibility concerns
[2]	Imaging-genetics/GWAS	Genetic susceptibility in AD	Imaging-genetic associations	*APOE*- and GWAS-associated phenotypes	Integrative computational approaches	Identification of genetic loci associated with disease heterogeneity	Primarily correlational findings
[27]	AI-driven multi-omics integration	AD multi-omics integration	Imaging and genomic datasets	Multi-omics-derived imaging signatures	AI-based integrative frameworks	Potential linkage between molecular pathways and imaging phenotypes	Limited longitudinal validation

Abbreviations: AD—Alzheimer’s disease; AI—artificial intelligence; APOE = apolipoprotein E; CNN—convolutional neural network; GWAS = genome-wide association study/studies; ML—machine learning; MRI—magnetic resonance imaging; PET—positron emission tomography; SPECT—single-photon emission computed tomography.

## Data Availability

No new data were generated or analyzed in this study.

## Abbreviations

The following abbreviations are used in this manuscript:

AD	Alzheimer’s disease
APOE	Apolipoprotein E
APP	Amyloid precursor protein
Aβ	Amyloid-β
CLU	Clusterin
CR1	Complement receptor 1
EOFAD	Early-onset familial Alzheimer’s disease
GWAS	Genome-wide association studies
IBSI	Image Biomarker Standardization Initiative
LOAD	Late-onset Alzheimer’s disease
MAPT	Microtubule-associated protein tau
ML	Machine learning
MRI	Magnetic resonance imaging
PET	Positron emission tomography
PICALM	Phosphatidylinositol binding clathrin assembly protein
PSEN1/PSEN2	Presenilin 1 or 2
RF	Random forests
ROIs	Regions of interest
SUVRs	Standardized uptake value ratios
SVM	Support vector machines
TREM2	Triggering receptor expressed on myeloid cells 2

## References

[1] Karch C.M., Goate A.M. (2015). Alzheimer’s Disease Risk Genes and Mechanisms of Disease Pathogenesis. Biol. Psychiatry.

[2] Bellenguez C., Küçükali F., Jansen I.E., Kleineidam L., Moreno-Grau S., Amin N., Naj A.C., Campos-Martin R., Grenier-Boley B., Andrade V. (2022). New Insights into the Genetic Etiology of Alzheimer’s Disease and Related Dementias. Nat. Genet..

[3] Jin S.C., Benitez B.A., Karch C.M., Cooper B., Skorupa T., Carrell D., Norton J.B., Hsu S., Harari O., Cai Y. (2014). Coding Variants in TREM2 Increase Risk for Alzheimer’s Disease. Hum. Mol. Genet..

[4] Long J.M., Holtzman D.M. (2019). Alzheimer Disease: An Update on Pathobiology and Treatment Strategies. Cell.

[5] Wilson D.M., Cookson M.R., Van Den Bosch L., Zetterberg H., Holtzman D.M., Dewachter I. (2023). Hallmarks of Neurodegenerative Diseases. Cell.

[6] Lambert J.C., Ibrahim-Verbaas C.A., Harold D., Naj A.C., Sims R., Bellenguez C., DeStafano A.L., Bis J.C., Beecham G.W., Grenier-Boley B. (2013). Meta-Analysis of 74,046 Individuals Identifies 11 New Susceptibility Loci for Alzheimer’s Disease. Nat. Genet..

[7] Jay T.R., von Saucken V.E., Landreth G.E. (2017). TREM2 in Neurodegenerative Diseases. Mol. Neurodegener..

[8] Jiang T., Yu J.-T., Zhu X.-C., Tan L. (2013). TREM2 in Alzheimer’s Disease. Mol. Neurobiol..

[9] Rusek M., Smith J., El-Khatib K., Aikins K., Czuczwar S.J., Pluta R. (2023). The Role of the JAK/STAT Signaling Pathway in the Pathogenesis of Alzheimer’s Disease: New Potential Treatment Target. Int. J. Mol. Sci..

[10] Heneka M.T., Carson M.J., El Khoury J., Landreth G.E., Brosseron F., Feinstein D.L., Jacobs A.H., Wyss-Coray T., Vitorica J., Ransohoff R.M. (2015). Neuroinflammation in Alzheimer’s Disease. Lancet Neurol..

[11] Hardy J.A., Higgins G.A. (1992). Alzheimer’s Disease: The Amyloid Cascade Hypothesis. Science.

[12] Wang Y., Mandelkow E. (2016). Tau in Physiology and Pathology. Nat. Rev. Neurosci..

[13] McManus R.M., Heneka M.T. (2017). Role of Neuroinflammation in Neurodegeneration: New Insights. Alzheimers Res. Ther..

[14] Heneka M.T., Kummer M.P., Latz E. (2014). Innate Immune Activation in Neurodegenerative Disease. Nat. Rev. Immunol..

[15] Parekh V., Jacobs M.A. (2016). Radiomics: A New Application from Established Techniques. Expert Rev. Precis. Med. Drug Dev..

[16] Gillies R.J., Kinahan P.E., Hricak H. (2016). Radiomics: Images Are More than Pictures, They Are Data. Radiology.

[17] Lambin P., Rios-Velazquez E., Leijenaar R., Carvalho S., van Stiphout R.G.P.M., Granton P., Zegers C.M.L., Gillies R., Boellard R., Dekker A. (2012). Radiomics: Extracting More Information from Medical Images Using Advanced Feature Analysis. Eur. J. Cancer.

[18] Liu Y., Zhao S., Wu Z., Liang H., Chen X., Huang C., Lu H., Yuan M., Xue X., Luo C. (2023). Virtual Biopsy Using CT Radiomics for Evaluation of Disagreement in Pathology between Endoscopic Biopsy and Postoperative Specimens in Patients with Gastric Cancer: A Dual-Energy CT Generalizability Study. Insights Imaging.

[19] Khalili N., Kazerooni A.F., Familiar A., Haldar D., Kraya A., Foster J., Koptyra M., Storm P.B., Resnick A.C., Nabavizadeh A. (2023). Radiomics for Characterization of the Glioma Immune Microenvironment. npj Precis. Oncol..

[20] Riberdy V., Guida A., Rioux J., Brewer K. (2025). Radiomics in Preclinical Imaging Research: Methods, Challenges and Opportunities. npj Imaging.

[21] McCague C., Ramlee S., Reinius M., Selby I., Hulse D., Piyatissa P., Bura V., Crispin-Ortuzar M., Sala E., Woitek R. (2023). Introduction to Radiomics for a Clinical Audience. Clin. Radiol..

[22] Mishra A.K., Jain S. (2026). Integrative Multiomics Insights into the Genetic and Epigenetic Architecture of Alzheimer’s Disease. ACS Chem. Neurosci..

[23] Cardillo M., Katam K., Suravajhala P. (2025). Advancements in Multi-Omics Research to Address Challenges in Alzheimer’s Disease: A Systems Biology Approach Utilizing Molecular Biomarkers and Innovative Strategies. Front. Aging Neurosci..

[24] Jack C.R.J., Bennett D.A., Blennow K., Carrillo M.C., Dunn B., Haeberlein S.B., Holtzman D.M., Jagust W., Jessen F., Karlawish J. (2018). NIA-AA Research Framework: Toward a Biological Definition of Alzheimer’s Disease. Alzheimers Dement..

[25] Villemagne V.L., Doré V., Burnham S.C., Masters C.L., Rowe C.C. (2018). Imaging Tau and Amyloid-β Proteinopathies in Alzheimer Disease and Other Conditions. Nat. Rev. Neurol..

[26] Villemagne V.L., Burnham S., Bourgeat P., Brown B., Ellis K.A., Salvado O., Szoeke C., Macaulay S.L., Martins R., Maruff P. (2013). Amyloid β Deposition, Neurodegeneration, and Cognitive Decline in Sporadic Alzheimer’s Disease: A Prospective Cohort Study. Lancet Neurol..

[27] Ren F., Wei J., Chen Q., Hu M., Yu L., Mi J., Zhou X., Qin D., Wu J., Wu A. (2025). Artificial Intelligence-Driven Multi-Omics Approaches in Alzheimer’s Disease: Progress, Challenges, and Future Directions. Acta Pharm. Sin. B.

[28] Ułamek-Kozioł M., Furmaga-Jabłońska W., Januszewski S., Brzozowska J., Sciślewska M., Jabłoński M., Pluta R. (2013). Neuronal Autophagy: Self-Eating or Self-Cannibalism in Alzheimer’s Disease. Neurochem. Res..

[29] Ossenkoppele R., van der Kant R., Hansson O. (2022). Tau Biomarkers in Alzheimer’s Disease: Towards Implementation in Clinical Practice and Trials. Lancet Neurol..

[30] Tanaka M., Saito S., Inoue T., Satoh-Asahara N., Ihara M. (2020). Potential Therapeutic Approaches for Cerebral Amyloid Angiopathy and Alzheimer’s Disease. Int. J. Mol. Sci..

[31] Pluta R., Ułamek-Kozioł M., Huang X. (2020). Tau Protein-Targeted Therapies in Alzheimer’s Disease: Current State and Future Perspectives. Molecular Biology of Dementia.

[32] Stoothoff W.H., Johnson G.V.W. (2005). Tau Phosphorylation: Physiological and Pathological Consequences. Biochim. Biophys. Acta.

[33] Mueller R.L., Combs B., Alhadidy M.M., Brady S.T., Morfini G.A., Kanaan N.M. (2021). Tau: A Signaling Hub Protein. Front. Mol. Neurosci..

[34] Groot C., Villeneuve S., Smith R., Hansson O., Ossenkoppele R. (2022). Tau PET Imaging in Neurodegenerative Disorders. J. Nucl. Med..

[35] Rubio-Perez J.M., Morillas-Ruiz J.M. (2012). A Review: Inflammatory Process in Alzheimer’s Disease, Role of Cytokines. Sci. World J..

[36] Clark L.F., Kodadek T. (2016). The Immune System and Neuroinflammation as Potential Sources of Blood-Based Biomarkers for Alzheimer’s Disease, Parkinson’s Disease, and Huntington’s Disease. ACS Chem. Neurosci..

[37] Latta C.H., Brothers H.M., Wilcock D.M. (2015). Neuroinflammation in Alzheimer’s Disease; a Source of Heterogeneity and Target for Personalized Therapy. Neuroscience.

[38] Jain M., Singh M.K., Shyam H., Mishra A., Kumar S., Kumar A., Kushwaha J. (2021). Role of JAK/STAT in the Neuroinflammation and Its Association with Neurological Disorders. Ann. Neurosci..

[39] Wang H., Shen Y., Chuang H., Chiu C., Ye Y., Zhao L. (2019). Neuroinflammation in Alzheimer’s Disease: Microglia, Molecular Participants and Therapeutic Choices. Curr. Alzheimer Res..

[40] Mondragón-Rodríguez S., Perry G., Zhu X., Moreira P.I., Acevedo-Aquino M.C., Williams S. (2013). Phosphorylation of Tau Protein as the Link between Oxidative Stress, Mitochondrial Dysfunction, and Connectivity Failure: Implications for Alzheimer’s Disease. Oxid. Med. Cell. Longev..

[41] Scheff S.W., Price D.A., Schmitt F.A., Scheff M.A., Mufson E.J. (2011). Synaptic Loss in the Inferior Temporal Gyrus in Mild Cognitive Impairment and Alzheimer’s Disease. J. Alzheimers Dis..

[42] Wood J.I., Dulewicz M., Ge J., Stringer K., Szadziewska A., Desai S., Koutarapu S., Hajar H.B., Fenson L., Blennow K. (2025). Isotope Encoded Spatial Biology Identifies Amyloid Plaque-Age-Dependent Structural Maturation, Synaptic Loss, and Increased Toxicity. Nat. Commun..

[43] Wang J., Jin C., Zhou J., Zhou R., Tian M., Lee H.J., Zhang H. (2023). PET Molecular Imaging for Pathophysiological Visualization in Alzheimer’s Disease. Eur. J. Nucl. Med. Mol. Imaging.

[44] Mueller A., Bullich S., Barret O., Madonia J., Berndt M., Papin C., Perrotin A., Koglin N., Kroth H., Pfeifer A. (2020). Tau PET Imaging with [18F]PI-2620 in Patients with Alzheimer Disease and Healthy Controls: A First-in-Humans Study. J. Nucl. Med..

[45] Frisoni G.B., Fox N.C., Jack C.R., Scheltens P., Thompson P.M. (2010). The Clinical Use of Structural MRI in Alzheimer Disease. Nat. Rev. Neurol..

[46] Rajalakshmi M., Karthik C. (2024). A Deep Learning-Based Framework for Alzheimer’s Disease Diagnosis and Progression Prediction from MRI Images. ICTACT J. Image Video Process..

[47] Young P.N.E., Estarellas M., Coomans E., Srikrishna M., Beaumont H., Maass A., Venkataraman A.V., Lissaman R., Jiménez D., Betts M.J. (2020). Imaging Biomarkers in Neurodegeneration: Current and Future Practices. Alzheimers Res. Ther..

[48] DeTure M.A., Dickson D.W. (2019). The Neuropathological Diagnosis of Alzheimer’s Disease. Mol. Neurodegener..

[49] Banzo I., Jiménez-Bonilla J., Ortega-Nava F., Quirce R., Martínez-Rodríguez I., de Arcocha-Torres M., Rodríguez E., Vázquez J.L., Sánchez P.J., Martínez-Amador N. (2014). Amyloid Imaging with 11C-PIB PET/CT and Glucose Metabolism with 18F-FDG PET/CT in a Study on Cognitive Impairment in the Clinical Setting. Nucl. Med. Commun..

[50] Guedj E., Varrone A., Boellaard R., Albert N.L., Barthel H., van Berckel B., Brendel M., Cecchin D., Ekmekcioglu O., Garibotto V. (2022). EANM Procedure Guidelines for Brain PET Imaging Using [18F]FDG, Version 3. Eur. J. Nucl. Med. Mol. Imaging.

[51] Clark C.M., Schneider J.A., Bedell B.J., Beach T.G., Bilker W.B., Mintun M.A., Pontecorvo M.J., Hefti F., Carpenter A.P., Flitter M.L. (2011). Use of Florbetapir-PET for Imaging β-Amyloid Pathology. JAMA.

[52] Leuzy A., Chiotis K., Lemoine L., Gillberg P.-G., Almkvist O., Rodriguez-Vieitez E., Nordberg A. (2019). Tau PET Imaging in Neurodegenerative Tauopathies—Still a Challenge. Mol. Psychiatry.

[53] Burkett B.J., Johnson D.R., Lowe V.J. (2024). Evaluation of Neurodegenerative Disorders with Amyloid-β, Tau, and Dopaminergic PET Imaging: Interpretation Pitfalls. J. Nucl. Med..

[54] Hall B., Mak E., Cervenka S., Aigbirhio F.I., Rowe J.B., O’Brien J.T. (2017). In Vivo Tau PET Imaging in Dementia: Pathophysiology, Radiotracer Quantification, and a Systematic Review of Clinical Findings. Ageing Res. Rev..

[55] Tiepolt S., Patt M., Aghakhanyan G., Meyer P.M., Hesse S., Barthel H., Sabri O. (2019). Current Radiotracers to Image Neurodegenerative Diseases. EJNMMI Radiopharm. Chem..

[56] Zhang S., Wang X., Gao X., Chen X., Li L., Li G., Liu C., Miao Y., Wang R., Hu K. (2025). Radiopharmaceuticals and Their Applications in Medicine. Signal Transduct. Target. Ther..

[57] Pokrzyk J., Kulczyńska-Przybik A., Guzik-Makaruk E., Winkel I., Mroczko B. (2025). Clinical Importance of Amyloid Beta Implication in the Detection and Treatment of Alzheimer’s Disease. Int. J. Mol. Sci..

[58] Fernandez-Alvarez M., Atienza M., Cantero J.L. (2023). Cortical Amyloid-Beta Burden Is Associated with Changes in Intracortical Myelin in Cognitively Normal Older Adults. Transl. Psychiatry.

[59] Dhoundiyal S., Srivastava S., Kumar S., Singh G., Ashique S., Pal R., Mishra N., Taghizadeh-Hesary F. (2024). Radiopharmaceuticals: Navigating the Frontier of Precision Medicine and Therapeutic Innovation. Eur. J. Med. Res..

[60] Chen P.-H., Hsu J.-L. (2026). Alzheimer’s Disease Diagnosis: An Update and Review of Biomarkers, Positron Emission Tomography, and Emerging Therapies. J. Formos. Med. Assoc..

[61] D’Anna L., Searle G., Harvey K., Matthews P.M., Veltkamp R. (2023). Time Course of Neuroinflammation after Human Stroke—A Pilot Study Using Co-Registered PET and MRI. BMC Neurol..

[62] Li A., Lian J., Vardhanabhuti V. (2025). Multi-Modal Machine Learning Approach for Early Detection of Neurodegenerative Diseases Leveraging Brain MRI and Wearable Sensor Data. PLoS Digit. Health.

[63] Rabinovici G.D., Knopman D.S., Arbizu J., Benzinger T.L.S., Donohoe K.J., Hansson O., Herscovitch P., Kuo P.H., Lingler J.H., Minoshima S. (2025). Updated Appropriate Use Criteria for Amyloid and Tau PET: A Report from the Alzheimer’s Association and Society for Nuclear Medicine and Molecular Imaging Workgroup. J. Nucl. Med..

[64] Phang K.A.S., Tan C.H., Alzheimer’s Disease Neuroimaging Initiative (2025). Cognitive Variation Reflects Amyloid, Tau, and Neurodegenerative Biomarkers in Alzheimer’s Disease. Geroscience.

[65] Zilioli A., Mohanty R., Rosenberg A., Matton A., Granberg T., Hagman G., Spallazzi M., Ferreira D., Kivipelto M., Westman E. (2026). MRI-Based Atrophy Subtypes in a Young Memory Clinic Cohort: Associations with Clinical and Biomarker Profiles. Alzheimers Res. Ther..

[66] Fürtjes A.E., Foote I.F., Xia C., Davies G., Moodie J., Taylor A., Liewald D.C., Redmond P., Corley J., McIntosh A.M. (2025). Measurement Characteristics and Genome-Wide Correlates of Lifetime Brain Atrophy Estimated from a Single MRI. Nat. Commun..

[67] Tsougos I., Valotassiou V., Tsivaka D., Satra M., Angelidis G., Papatriantafyllou J., Panagiotidis E., Dardiotis E., Hadjigeorgiou G., Georgoulias P. (2026). SPECT and PET Imaging of Alzheimer’s Disease Revisited: From Biomarkers to Artificial Intelligence-Based Prediction. Ann. Nucl. Med..

[68] van Griethuysen J.J.M., Fedorov A., Parmar C., Hosny A., Aucoin N., Narayan V., Beets-Tan R.G.H., Fillion-Robin J.-C., Pieper S., Aerts H.J.W.L. (2017). Computational Radiomics System to Decode the Radiographic Phenotype. Cancer Res..

[69] Hao X., Li Y., Wang X., Ma C., Liu R., Jiao Y., Dong C., Liu J. (2025). Multimodal Radiomics of Cerebellar Subregions for Machine Learning-Driven Alzheimer’s Disease Diagnosis. Front. Aging Neurosci..

[70] Mayerhoefer M.E., Materka A., Langs G., Häggström I., Szczypiński P., Gibbs P., Cook G. (2020). Introduction to Radiomics. J. Nucl. Med..

[71] Xiao H., He X., Zhou W., Guo X., Cai X., Li T. (2025). The Application of Radiomics in the Diagnosis and Evaluation of Cognitive Impairment Related to Neurological Diseases. Front. Neurosci..

[72] Yu Z., Mulholland A., Huang T., Liu Q. (2026). Multimodal AI for Alzheimer Disease Diagnosis: Systematic Review of Datasets, Models, and Modalities. J. Med. Internet Res..

[73] Zarei O., Talebi Moghaddam M., Moradi Vastegani S. (2026). Machine Learning and Deep Learning in Clinical Practice: Advancing Neurodegenerative Disease Diagnosis with Multimodal Markers. Brain Res. Bull..

[74] Aguirre-Meneses H., Stoehr-Muñoz P., Molina-Gonzalez M., Nuñez-Gaona M.-A., Roldan-Valadez E. (2025). Radiomics and the Image Biomarker Standardisation Initiative (IBSI): A Narrative Review Using a Six-Question Map and Implementation Framework for Reproducible Imaging Biomarkers. Cureus.

[75] Xu Y., Li Y., Wang F., Zhang Y., Huang D. (2025). Addressing the Current Challenges in the Clinical Application of AI-Based Radiomics for Cancer Imaging. Front. Med..

[76] Amr Y., Gad W., Leiva V., Martin-Barreiro C., Abdelkader T. (2026). Comparative Analysis of Supervised and Ensemble Models with Unsupervised Exploration for Alzheimer’s Disease Prediction. Sci. Rep..

[77] Campagner A., Marconi L., Bianchi E., Arosio B., Rossi P., Annoni G., Lucchi T.A., Montano N., Cabitza F. (2025). Uncovering Hidden Subtypes in Dementia: An Unsupervised Machine Learning Approach to Dementia Diagnosis and Personalization of Care. J. Biomed. Inform..

[78] Trezza A., Visibelli A., Roncaglia B., Spiga O., Santucci A. (2024). Unsupervised Learning in Precision Medicine: Unlocking Personalized Healthcare through AI. Appl. Sci..

[79] Hu T., Mousavirad S.J., Afsharizadeh M., O’Nils M. (2026). Deep Learning for MRI-Based Neurological Disease Diagnosis: A Comprehensive Survey of Advances, Challenges, and Benchmarks. Int. J. Comput. Intell. Syst..

[80] Mariotti F., Agostini A., Borgheresi A., Marchegiani M., Zannotti A., Giacomelli G., Pierpaoli L., Tola E., Galiffa E., Giovagnoni A. (2025). Insights into Radiomics: A Comprehensive Review for Beginners. Clin. Transl. Oncol..

[81] Avanzo M., Soda P., Bertolini M., Bettinelli A., Rancati T., Stancanello J., Rampado O., Pirrone G., Drigo A. (2026). Robust Radiomics: A Review of Guidelines for Radiomics in Medical Imaging. Front. Radiol..

[82] Pudjihartono N., Fadason T., Kempa-Liehr A.W., O’Sullivan J.M. (2022). A Review of Feature Selection Methods for Machine Learning-Based Disease Risk Prediction. Front. Bioinform..

[83] Alemu R., Sharew N.T., Arsano Y.Y., Ahmed M., Tekola-Ayele F., Mersha T.B., Amare A.T. (2025). Multi-Omics Approaches for Understanding Gene-Environment Interactions in Noncommunicable Diseases: Techniques, Translation, and Equity Issues. Hum. Genom..

[84] Mahley R.W., Weisgraber K.H., Huang Y. (2006). Apolipoprotein E4: A Causative Factor and Therapeutic Target in Neuropathology, Including Alzheimer’s Disease. Proc. Natl. Acad. Sci. USA.

[85] Neu S.C., Pa J., Kukull W., Beekly D., Kuzma A., Gangadharan P., Wang L.-S., Romero K., Arneric S.P., Redolfi A. (2017). Apolipoprotein E Genotype and Sex Risk Factors for Alzheimer Disease: A Meta-Analysis. JAMA Neurol..

[86] Mashhour M.A., Youssef I., Wahed M.A., Mabrouk M.S. (2025). The Intersection of Genetics and Neuroimaging: A Systematic Review of Imaging Genetics in Neurological Disease for Personalized Treatment. J. Mol. Neurosci..

[87] Reitz C., Mayeux R. (2014). Alzheimer Disease: Epidemiology, Diagnostic Criteria, Risk Factors and Biomarkers. Biochem. Pharmacol..

[88] Burkett B.J., Bartlett D.J., McGarrah P.W., Lewis A.R., Johnson D.R., Berberoğlu K., Pandey M.K., Packard A.T., Halfdanarson T.R., Hruska C.B. (2023). A Review of Theranostics: Perspectives on Emerging Approaches and Clinical Advancements. Radiol. Imaging Cancer.

[89] Sarabia-Vallejo Á., López-Alvarado P., Menéndez J.C. (2023). Small-Molecule Theranostics in Alzheimer’s Disease. Eur. J. Med. Chem..

[90] Urbain J.-L., Scott A.M., Lee S.T., Buscombe J., Weston C., Hatazawa J., Kinuya S., Singh B., Haidar M., Ross A. (2023). Theranostic Radiopharmaceuticals: A Universal Challenging Educational Paradigm in Nuclear Medicine. J. Nucl. Med..

